# Quality of life and functional recovery after venous stent placement for postthrombotic syndrome: insights from the ARIVA multinational randomized trial

**DOI:** 10.1016/j.rpth.2026.106828

**Published:** 2026-07-10

**Authors:** Stefano Barco, Simon Wolf, Tim Sebastian, Riccardo M. Fumagalli, Evy Micieli, Christian Erbel, Houman Jalaie, Frederikus A. Klok, Oliver Schlager, Nils Kucher

**Affiliations:** 1 Department of Vascular Medicine, University Hospital Zurich, University of Zurich, Zurich, Switzerland; 2 Center for Thrombosis and Hemostasis, University Medical Center of the Johannes Gutenberg University Mainz, Mainz, Germany; 3 Department of Medicine - Thrombosis and Hemostasis, Leiden University Medical Center, Leiden, the Netherlands; 4 Department of Cardiology, Angiology and Pneumology, Heidelberg University Hospital, Heidelberg, Germany; 5 Clinic of Vascular and Endovascular Surgery, Rhineland-Westphalian Technical University, Aachen, Germany; 6 Division of Angiology, Department of Medicine II, Medical University of Vienna, Vienna, Austria

**Keywords:** endovascular procedures, quality of life, postthrombotic syndrome, thrombosis

## Abstract

**Background:**

Postthrombotic syndrome (PTS) is a disabling complication of deep vein thrombosis and is associated with impaired functional status.

**Objectives:**

To assess the impact of stent placement for post-thrombotic syndrome on functional outcomes and quality of life.

**Methods:**

The Aspirin Plus Rivaroxaban Versus Rivaroxaban Alone for the Prevention of Venous Stent Thrombosis Among Patients with Postthrombotic Syndrome trial was a multinational, randomized study comparing rivaroxaban plus aspirin vs rivaroxaban monotherapy after venous revascularization for PTS. The Villalta score and the Chronic Venous Insufficiency Quality of Life Questionnaire-20 (CIVIQ-20) were assessed at randomization and during 6-month follow-up. A multivariable mixed-effects model was used to identify predictors of quality of life (QoL).

**Results:**

Of 149 enrolled patients, 55% had moderate-to-severe PTS at baseline, according to the Villalta score. Compared with patients with mild PTS, they were characterized by longer stent segments, higher body mass index, and were more frequently smokers. The 6-month primary patency rate was similar between groups: 91.5% vs 95.5%, respectively. The improvement in PTS severity consisted of an overall mean reduction of −6.5 (SD 4.6) points, corresponding to a change of −8.5 (5.0) points among patients with moderate-to-severe PTS and −4 (2.6) points among patients with mild PTS after 6 months. Median CIVIQ-20 scores were lower, indicating worse QoL, among patients with moderate-to-severe PTS compared with mild PTS, consistently at baseline and all follow-up timepoints. Female sex and greater PTS severity were independently associated with worse QoL after adjustment for age, body mass index, treatment group, adverse events, and timepoint.

**Conclusion:**

Over the 6 months after endovascular treatment accessory to standard care for PTS, both PTS severity measured by the Villalta score and QoL measured by CIVIQ-20 improved. However, greater PTS severity and female sex were independently associated with worse QoL outcomes.

## Essentials

• Patients with (vs without) postthrombotic syndrome (PTS) have worse quality of life.
• Post hoc analysis of the ARIVA trial with a focus on changes in quality of life.
• Successful endovascular treatment for PTS may lead to improvements in quality of life.
• Patients with more severe PTS and women had worse quality of life despite revascularization.

## 1 Introduction

Postthrombotic syndrome (PTS) is a chronic and debilitating condition, developing in up to 30% to 50% of patients with iliofemoral deep vein thrombosis, despite standard anticoagulation and compression therapy [1,2]. The condition is characterized by persistent pain, swelling, skin changes, venous claudication, and, in severe cases, ulcers [1]. Patients with PTS are characterized by worse quality of life (QoL) than those without PTS [3,4]. This may be due to functional limitations that reduce mobility and daily activity levels as well as because of symptom burden such as pain. Moreover, chronic ulcers may result in anxiety and depression, and prior thrombosis may substantially affect the emotional well-being and psychosocial functioning of patients [[5], [6], [7]].

It has been shown that the degree of PTS severity correlates with QoL, so improving PTS-related symptoms may lead to better functional outcomes [7,8]. Conservative management with compression therapy, physical rehabilitation, and pharmacologic treatment aims to alleviate symptoms, but their impact on QoL and functional outcomes has been poorly studied. Recently, the results of a randomized controlled trial showed that endovascular treatment accessory to standard PTS care can improve symptom relief and QoL compared with conservative treatment alone [9]. As the predictors of PTS course and QoL after endovascular treatment are unknown, it is important to follow these not only for stent patency but also focusing on functional outcomes and QoL.

The Aspirin Plus Rivaroxaban Versus Rivaroxaban Alone for the Prevention of Venous Stent Thrombosis Among Patients with Postthrombotic Syndrome (ARIVA) trial demonstrated that combining aspirin with rivaroxaban did not appear to prevent stent thrombosis compared with rivaroxaban monotherapy in patients with PTS undergoing venous revascularization [10]. In ARIVA, functional scales and QoL were measured at predefined timepoints at baseline and over 6 months. In this post hoc analysis, we stratified patients according to the degree of PTS severity and studied the course and predictors of functional outcomes and QoL after venous revascularization.

## 2 Methods

The ARIVA trial (EudraCT 2019-001723-12, ClinicalTrials.gov NCT04128956) was an open-label, multicenter, randomized phase 3 trial with independently adjudicated clinical outcomes that enrolled patients who had undergone venous stent placement for PTS [10]. Patients were included at 6 centers in 3 countries (Austria, Switzerland, and Germany). The sponsor of the study was the University of Zurich. Institutional review committees approved the study, and all subjects gave informed consent before enrollment.

Adults aged ≤75 years with a confirmed diagnosis of PTS (Villalta score >4) due to a documented stenosis or occlusion of the inferior vena cava, iliac vein, or common femoral vein were eligible. Major exclusion criteria were active cancer, contraindications to antithrombotic therapy, requirement for anticoagulants other than rivaroxaban, renal/hepatic dysfunction, or the presence of an acute venous thrombosis. After technically successful venous stent intervention, they were randomized provided they had no contraindications to antithrombotic therapy or contraindications to rivaroxaban. Eligible patients were randomly assigned after successful stent placement to receive either aspirin 100 mg once daily plus rivaroxaban 20 mg once daily or rivaroxaban 20 mg once daily as a stand-alone treatment in a 1:1 ratio. All patients received standard of care treatment, including the recommendation to wear class II compression stockings (pressure of 23 to 32 mmHg) for a period of at least 6 months and wound management, if necessary.

The primary efficacy outcome was the composite of freedom from occlusion in the treated segment and reintervention needed to maintain patency (primary patency rate) within 6 months, as routinely assessed with duplex ultrasound of the affected leg according to a standardized protocol at 3 and 6 months. Secondary efficacy outcomes included radiological patency parameters as well as QoL (chronic venous insufficiency QoL questionnaire, CIVIQ-20) and changes in PTS functional scores, notably the Villalta score and the revised venous clinical severity score (rVCSS) at 3 and 6 months. The CIVIQ-20 is a patient-reported assessment scale consisting of 20 items across 4 dimensions: psychological (9 items), physical (4 items), social (3 items), and pain (4 items). Patients completed the questionnaire themselves, without assistance from the clinical team. Each item was rated on a 5-point scale from 1 (best possible) to 5 (worst) [11]. The Villalta score for the affected leg consists of 5 patient-rated venous symptoms and 6 clinician-rated physical signs, which are each rated on a 4-point scale (from 0, none, to 3, severe) [12]. The rVCSS for the affected leg consists of 10 venous symptoms, which are each rated on a 4-point scale from 0 (none) to 3 (severe) and summed [13].

The present analysis was conducted on a complete-case dataset for baseline functional data (Villalta score). This approach was preferred over missing data imputation methods due to the relatively small sample size of the trial, as the imputation model would not perform well to accurately predicting missing values and may introduce bias. In the descriptive analysis, data are presented either as count and percentage or, for continuous variables, as median and IQR. Comparison of groups was performed by calculating the absolute risk difference with corresponding 2-sided 95% CIs of the treatment difference. To test the effect of the intervention on Villalta score and QoL from baseline to last visit, a Wilcoxon signed rank test was performed. A multivariable mixed-effects model was used to identify predictors of QoL, adjusting for variables identified based on their clinical relevance and potential association with QoL based on prior studies from the literature. Adverse events, including stent-thrombosis, recurrent venous thromboembolism, and bleeding events, were included as time-varying variables, whereas for the other variable, the baseline value was entered into the model. The number needed to treat (NNT) was simulated based on varying CIVIQ-20 reduction thresholds among the study population, since a clinically significant threshold has not been established. The proportion of patients with PTS without an intervention that reached a given threshold throughout a 6-month period was simulated based on a normal distribution with varying mean reduction and the SD found in our study. R (version 4.5.0; packages: *tidyverse*, *survival*, *lmer*) served for data analysis. A *P* value < .05 was considered statistically significant.

## 3 Results

A total of 149 (92%) patients of a total of 162 patients from the full-analysis set had complete available data concerning the severity of PTS at baseline and 6-month data. At baseline, moderate-to-severe PTS according to the Villalta score was reported in 82 (55%) of 149 patients, whereas the remaining 67 (45%) patients had a mild PTS. Of 82 patients with moderate-to-severe PTS, 31 had a severe PTS and 11 had ulcerations.

Table 1 [15] provides an overview of the baseline and demographic characteristics stratified according to the severity of PTS. A few variables showed clinically relevant differences between groups. Patients with mild (vs moderate-to-severe) PTS were less frequently smokers (13% vs 37%) and had a shorter length of the postthrombotic venous segment, requiring a stent extension below the inguinal ligament in 51% of cases vs 70% of those with moderate-to-severe PTS. Furthermore, the median body mass index (BMI) was lower among patients with mild PTS (24.3 kg/m^2^; IQR, 22.5-28.4) than among those with moderate-to-severe PTS (25.9 kg/m^2^; IQR, 23.1-31.7).

**Table 1:** Baseline and demographic characteristics.

Characteristic	Mild PTS *N* = 67	Moderate-to-severe PTS *N* = 82	Overall *N* = 149
Age (y), median (IQR)	40 (29, 54)	40 (32, 54)	40 (31, 54)
Women, *n* (%)	44 (65.7)	49 (59.8)	93 (62.4)
Body mass index (kg/m^2^), median (IQR)	24.3 (22.5, 28.4)	25.9 (23.1, 31.7)	24.7 (22.9, 30.1)
Race and ethnic group, *n* (%)			
Caucasian	63 (96.9)	73 (97.3)	136 (97.1)
Black	0 (0.0)	2 (2.7)	2 (1.4)
Asian	2 (3.1)	0 (0.0)	2 (1.4)
Active smokers, *n* (%)	9 (13.4)	30 (37.0)	39 (26.4)
Ulcerative colitis, *n* (%)	3 (4.5)	0 (0.0)	3 (2.0)
Obesity, *n* (%)	3 (4.5)	5 (6.1)	8 (5.4)
Rheumatoid arthritis, *n* (%)	1 (1.5)	0 (0.0)	1 (0.7)
Hypothyroidism, *n* (%)	5 (7.5)	6 (7.3)	11 (7.4)
Side of index intervention, *n* (%)			
Bilateral	10 (14.9)	17 (20.7)	27 (18.1)
Right side only	6 (9.0)	2 (2.4)	8 (5.4)
Left side only	49 (73.1)	63 (76.8)	112 (75.2)
Endovascular inferior vena cava reconstruction, *n* (%)	14 (20.9)	14 (17.1)	28 (18.8)
Stent extension to the common femoral vein, femoral vein or deep femoral vein, *n* (%)	34 (50.7)	57 (69.5)	91 (61.1)
Jalaie anatomical classification of post-thrombotic syndrome extension, *n* (%)			
I	12 (17.9)	8 (9.8)	20 (13.4)
II	8 (11.9)	9 (11.0)	17 (11.4)
III	11 (16.4)	12 (14.6)	23 (15.4)
IVa	13 (19.4)	22 (26.8)	35 (23.5)
IVb	1 (1.5)	0	1 (0.7)
VI	10 (14.9)	15 (18.3)	25 (16.8)

The degree of postthrombotic syndrome (PTS) severity was assessed with the Villalta score. A score of 5-9 points signifies mild disease, 10-14 moderate disease, and ≥15 severe disease. The Jalaie anatomical classification of PTS has been proposed to group patients with PTS based on the extension of postthrombotic vein disease, with higher numbers signifying involvement of longer venous segments [14].

**Figure 1:**
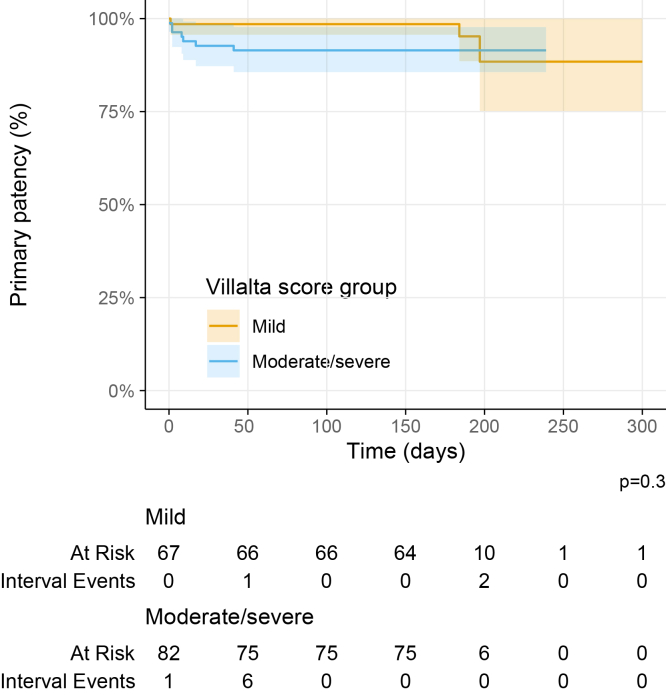
Primary patency among patients with moderate-to-severe postthrombotic syndrome (PTS) and mild PTS according to the Villalta score.

The primary patency rate after 6 months was higher among patients with mild (95.5%) vs moderate-to-severe PTS (91.5%); this difference did not reach statistical significance, with an absolute difference of 4.1% (95% CI. −3.8% to 11.9%; Table 2). A Kaplan–Meier curve estimator for the primary patency rate is depicted in Figure 1. The magnitude of PTS improvement according to the Villalta score depended on the initial degree of PTS severity. Patients with mild PTS exhibited an improvement of −4.0 (SD 2.6) points in the Villalta score. Patients with moderate-to-severe PTS exhibited an improvement of −8.5 (5.0) points in the Villalta score. The changes in rVCSS were −3.2 (3.1) and −4.7 (4.3) in the 2 groups, respectively.

**Table 2:** Patency rate and changes from baseline in functional status, symptom severity and quality-of-life scores, overall and according to PTS severity, at 6 months.

Characteristic	Overall *N* = 142	Mild PTS *N* = 67	Moderate/severe PTS *N* = 82	Group difference (95% CI)	*P* value
Primary patency rate after 6 mo, *n* (%)	132 (93.0)	64 (95.5)	75 (91.5)	4.1 (−3.8 to 11.9)	.3
Change in Villalta score after 6 mo (points), absolute mean reduction (SD)	−6.5 (4.6)	−4.0 (2.6)	−8.5 (5.0)	6.5 (3.2 to 5.8)	<.001
Change in rVCSS after 6 mo (points), absolute mean reduction (SD)	−4.0 (3.9)	−3.2 (3.1)	−4.7 (4.3)	1.5 (0.3 to 2.7)	.02
Change in CIVIQ-20 after 6 mo (points), absolute mean reduction (SD)	−18.7 (17.8)	−15.6 (19.5)	−21.3 (15.8)	5.7 (−0.2 to 11.6)	.06

Change in Villalta score and rVCSS includes all patients with measured values for baseline and V3. Wilcoxon rank sum test.

CIVIQ-20, Chronic Venous Insufficiency Quality of Life Questionnaire-20; PTS, postthrombotic syndrome; rVCSS, revised venous clinical severity score.

**Figure 2:**
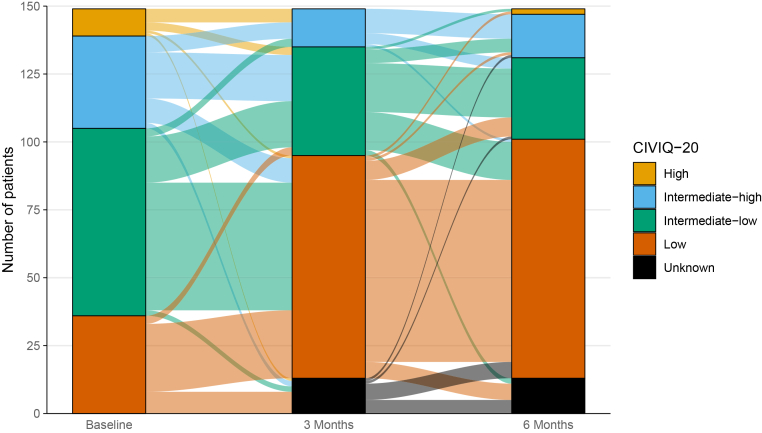
Time course of Chronic Venous Insufficiency Quality of Life Questionnaire-20 (CIVIQ-20) score at baseline, 3 months, and 6 months. Patients are grouped based on their CIVIQ-20 score at each timepoint. Lower CIVIQ-20 scores indicate better quality of life. High CIVIQ-20 (75-100 points), intermediate-high CIVIQ-20 (50-74 points), intermediate-low CIVIQ-20 (25-49 points), low CIVIQ-20 (0-24 points).

**Figure 3:**
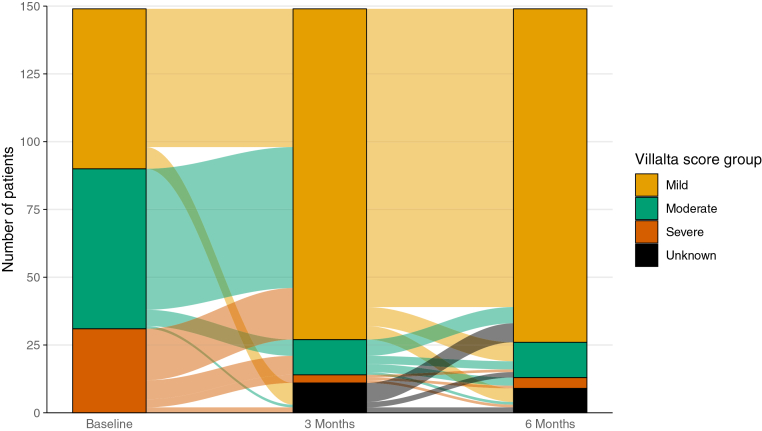
Time course of Villalta score group at baseline, 3 months, and 6 months. Patients are grouped based on their postthrombotic syndrome severity as per Villalta score at each timepoint.

The number of patients with the best QoL, as indicated by a CIVIQ-20 score of 0 to 25 of 100 points, increased from 31 (21%) at baseline to 86 (58%) after 3 months and 93 (62%) after 6 months at the last follow-up visit. At 6 months, the remaining patients had an intermediate-low score (*n* = 30; 20%), an intermediate-high score (*n* = 15; 11%), or a high score (*n* = 2; 1%). This corresponded to a significant decrease in the CIVIQ-20 score from baseline to last follow-up (*P* < .001). The course of the CIVIQ-20 score is depicted in Figure 2, whereas the median CIVIQ-20 score for each of the 20 items composing the overall score is depicted in the Supplementary Figure S1. The number of patients with moderate-to-severe Villalta score decreased from 90 (60%) at baseline to 16 (11%) after 3 months and 17 (11%) after 6 months (Figure 3). This corresponded to a significant decrease in the Villalta score from baseline to last follow-up (*P* < .001).

**Figure 4:**
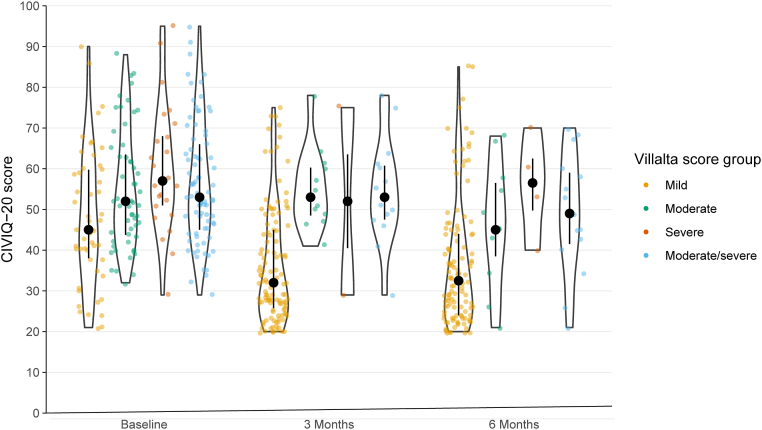
Median Chronic Venous Insufficiency Quality of Life Questionnaire-20 (CIVIQ-20) values stratified by Villalta score group at each visit. Patients are grouped based on their Villalta score at each timepoint: baseline (V1), month 3 (V2), and month 6 (V3). The black dot represents the median for each stratum, the vertical black lines the corresponding IQR.

Figure 4 displays the median CIVIQ-20 score across Villalta score groups at different timepoints. At any timepoint, patients with moderate-to-severe PTS displayed poorer QoL (CIVIQ-20) when compared to patients with mild PTS, as analyzed with nonparametric statistics. At baseline, patients with moderate-to-severe PTS had a median CIVIQ-20 score of 54 (IQR, 47-67) points vs 46 (IQR, 39.5-60) points among those with mild PTS. At 3 months, patients with moderate-to-severe PTS had a median score of 53 (IQR, 47.5-60.8) points vs 34 (IQR, 27-46) points among those with mild PTS. At 6 months, patients with moderate-to-severe PTS had a median score of 47 (IQR, 40.1-57.3) points vs 34 (IQR, 26-45) points among those with mild PTS.

Overall, 54.2% of patients had a reduction in CIVIQ-20 score of at least 15 points, and 77.1% had a reduction of at least 5 points. Supplementary Table S1 presents the simulated NNT for patients with PTS with (vs without) endovascular treatment accessory to standard PTS care to achieve an improvement in the CIVIQ-20 score and thus in QoL. Hereby, both the threshold in CIVIQ-20 score reduction among treated patients and the mean reduction over a 6-month period among a hypothetical untreated control population was calculated across a spectrum of values. The NNT ranged from 2.6 to 321.5, depending on the selected threshold based on rates among treated patients and on the estimated reduction in the hypothetical control population not receiving any endovascular intervention. In extreme cases with higher reduction in CIVIQ-20 score in the hypothetical control population without an intervention compared with patients with an intervention, the NNT became negative, signifying a potential negative influence of the intervention in these edge cases. We studied potential predictors of QoL during the follow-up. In a multivariable mixed regression model accounting for the time of assessment, female sex (9.5 points on CIVIQ-20 score; 95% CI, 3.5-15) and moderate-severe PTS (9.1 points; 95% CI, 3.8-14) were associated with higher CIVIQ-20 score (poorer QoL) after adjustment for the time of assessment, whereas BMI, age, intervention group, and occurrence of any adverse events during follow-up were not (Table 3).

**Table 3:** Multivariable mixed effects regression analysis of the association between clinical characteristics and overall quality of life at 6 months.

Independent variable	β (95% CI)
Age groups (y)	
≤30	Reference
30-40	0.55 (−6.8; 7.9)
>40	−1.2 (−8.0; 5.6)
Female sex (vs male sex)	9.5 (3.5; 15)
BMI groups (kg/m^2^)	
≤22.5	Reference
22.5-25	−1.2 (−8.5; 6.1)
>25	2.1 (−4.9; 9.2)
Moderate-to-severe PTS (vs mild PTS)	9.1 (3.8; 14)
Rivaroxaban-aspirin group (vs rivaroxaban monotherapy group)	−2.1 (−7.4; 3.2)
Any adverse event during follow-up (vs no events)	1.0 (−5.6; 7.6)
Visit 1	Reference
Visit 2 (after 3 mo)	−19 (−21; −16)
Visit 3 (after 6 mo)	−19 (−22; −16)

The β coefficient indicates the change in CIVIQ-20 points for each increase of one unit of the independent variable. Adverse events encompassed stent-related thrombosis and bleeding events.

BMI, body mass index; CIVIQ-20, Chronic Venous Insufficiency Quality of Life Questionnaire-20; PTS, postthrombotic syndrome.

## 4 Discussion

In this post hoc analysis of the randomized, multinational ARIVA trial, we studied the course of functional outcomes and QoL in patients with PTS after venous revascularization plus standard PTS care compared with baseline. Patients included in ARIVA reported clinical improvement in terms of PTS severity and patient-reported QoL over a 6-month follow-up period after endovascular treatment accessory to standard PTS care, in line with recent findings in a randomized controlled trial [9]. PTS severity and female sex emerged as significant independent predictors of poorer QoL, although the technical success and patency rate were overall very high.

Previous studies have studied the relationship between the severity of PTS and impairment in QoL, particularly among patients with chronic venous symptoms, restricted mobility, and ulceration [16,17]. In our study, we showed that the degree of improvement in Villalta score was greater among patients with moderate-to-severe (vs mild) PTS at baseline considering worse baseline status. A similar observation was seen in terms of improvement in QoL. However, despite symptom improvement, patients with moderate-severe PTS continued to report lower QoL at all timepoints. Stent patency rate was slightly higher among patients with mild PTS, possibly reflecting the shorter length of diseased venous segments; however, this difference was not statistically significant, suggesting that differences in QoL were not driven by technical failure but rather by other patient-level factors or unaccounted factors, such as persistent claudication or discomfort. Those are not encompassed in the Villalta score, as it primarily focuses on clinical signs and symptoms and may not fully capture functional limitations such as exercise tolerance. Incorporation of objective functional tests (eg, 6-minute or treadmill walk tests) may provide a more comprehensive assessment in future analyses [3].

Patients with more severe PTS had a higher BMI, more frequent history of smoking, and more extensive venous involvement, as evidenced by longer stented segments being needed and more frequent extension into the femoral and deep femoral veins. These factors might partly explain the more limited QoL gains observed in this subgroup. Finally, despite technical success, persistent inflammation and microvascular damage may underlie ongoing symptoms and impaired QoL in PTS patients, and comorbidities may influence QoL [18,19]. The observed improvement in patient-reported QoL may suggest that the hemodynamic improvement observed after venous revascularization for PTS accessory to standard PTS care translates not only into better functional outcomes (as per Villalta score) but also to improved individual perception and well-being. The fact that a considerable proportion continued to experience moderate-to-poor QoL calls for further research into adjunctive or supportive therapies, including psychological support in selected patients [[20], [21], [22]]. Of note, adverse events during follow-up did not influence QoL.

The association between female sex and poorer QoL outcomes remained after adjusting for baseline disease severity and other confounders. While the underlying reasons for this sex-based disparity are not fully understood, previous literature suggests that women may report greater symptom burden, higher pain perception, and more significant psychological impact in chronic vascular disease, such as pulmonary embolism, deep vein thrombosis, and myocardial infarctio [23,24]. Further studies are needed to explore whether sex-specific rehabilitation strategies or psychosocial interventions might improve recovery in this population. Moreover, studies to quantify the burden of PTS (and deep vein thrombosis) in terms of quality- or disability-adjusted life years are not available to date, and it is only recently that these have been quantified for the other manifestation of venous thromboembolism, pulmonary embolism [14].

The strengths of our study include the use of validated tools for assessing PTS severity and QoL, independent adjudication of patency outcomes, and the multicenter nature of the ARIVA trial conducted at 6 centers in 3 countries. Furthermore, we attempted to calculate the number of patients with PTS needed to treat with endovascular revascularization to improve QoL across the spectrum of cutoffs and expected reference values if no intervention is performed. This analysis could represent the basis for future trials powered for patient-reported outcomes. Some limitations must be acknowledged. First, this was a post hoc analysis, and while our findings are hypothesis-generating, they should be interpreted with caution. Second, our sample size was modest, which limited the power to detect small subgroup differences and precluded the use of imputation for missing data. Third, differences in Villalta score might partly reflect assessor bias. Fourth, change in QoL might partly reflect a patient’s expected improvement due to the performed intervention. Fifth, the study population was comprised of patients with technically successful stent placement; therefore, our results may overestimate the effect of endovascular treatment, as it cannot apply to patients in whom the intervention failed to achieve technical success. Last, the follow-up was limited to 6 months; longer-term data are needed to assess the durability of improvements and potential late complications.

In conclusion, technically successful endovascular treatment for PTS accessory to standard PTS care in patients with confirmed venous stenosis or occlusion may lead to improvement in symptom burden and QoL. However, patients with more severe PTS disease and female sex had worse QoL despite revascularization and standard of care medical treatment.
