# Supplementary material for: Quality of life and functional recovery after venous stent placement for postthrombotic syndrome: insights from the ARIVA multinational randomized trial
Source: Res Pract Thromb Haemost. 2026 Jul 10;10(5):106828. doi: 10.1016/j.rpth.2026.106828 (PMC13448022; doi:10.1016/j.rpth.2026.106828)
Supplement: Supplementary file 1 [file mmc1.docx]

**Supplemental Material**

*Supplementary Figure S1. Median score for each item of CIVIQ-20 stratified by visit*

*Supplementary Table S1. Number needed to treat for a reduction in CIVIQ-20 6 months after an intervention across CIVIQ-20 threshold*

| **CIVIQ-20 reduction threshold** | **0** | **5** | **10** | **15** | **20** |
| --- | --- | --- | --- | --- | --- |
| Proportion of patients in the study reaching the threshold (%) | 88.2 | 77.1 | 66.7 | 54.2 | 43.8 |
|  | | | | | |
| **Reduction in CIVIQ20 in patients with PTS without an intervention** | *Number needed to treat (NNT)* | | | | |
| **0** | 2.6 | 2.6 | 2.6 | 2.9 | 3.3 |
| **1** | 2.8 | 2.8 | 2.8 | 3.1 | 3.4 |
| **2** | 3.0 | 3.0 | 2.9 | 3.2 | 3.5 |
| **3** | 3.2 | 3.2 | 3.1 | 3.4 | 3.7 |
| **4** | 3.4 | 3.4 | 3.3 | 3.7 | 3.9 |
| **5** | 3.7 | 3.7 | 3.6 | 3.9 | 4.2 |
| **6** | 4.0 | 4.0 | 3.9 | 4.2 | 4.5 |
| **7** | 4.4 | 4.4 | 4.3 | 4.6 | 4.9 |
| **8** | 4.8 | 4.9 | 4.7 | 5.1 | 5.3 |
| **9** | 5.3 | 5.5 | 5.3 | 5.8 | 5.9 |
| **10** | 5.9 | 6.2 | 6.0 | 6.6 | 6.6 |
| **11** | 6.7 | 7.2 | 6.9 | 7.7 | 7.6 |
| **12** | 7.6 | 8.5 | 8.2 | 9.2 | 9.0 |
| **13** | 8.8 | 10.3 | 10.0 | 11.6 | 11.0 |
| **14** | 10.3 | 13.0 | 12.9 | 15.6 | 14.4 |
| **15** | 12.3 | 17.3 | 17.9 | 24.0 | 20.7 |

The number needed to treat (NNT) was estimated based on the proportion of patients reaching various thresholds. The proportion of patients with PTS without an intervention reaching the threshold was simulated based on a normal distribution with a varying mean and the standard deviation found for the improvement in CIVIQ-20 score in this study.
